# A Comparison of RNA Interference via Injection and Feeding in Honey Bees

**DOI:** 10.3390/insects13100928

**Published:** 2022-10-13

**Authors:** Yong Zhang, Zhen Li, Zi-Long Wang, Li-Zhen Zhang, Zhi-Jiang Zeng

**Affiliations:** 1 Honeybee Research Institute, Jiangxi Agricultural University, Nanchang 330045, China; 2 Jiangxi Province Key Laboratory of Honeybee Biology and Beekeeping, Nanchang 330045, China

**Keywords:** RNAi, siRNA, honey bee, brain

## Abstract

**Simple Summary:**

RNA interference is an important way to analyze gene function. It is also widely used in honey bees. It was thought that RNA interference with honey bee brain genes could only be achieved by injection. However, this method of injection is very complicated and can easily cause damage to bees. Recent studies have shown that specially treated siRNA can knockdown brain genes in animals by feeding them. Therefore, we used different approaches to deliver modified and unmodified siRNAs to investigate what methods can successfully interfere with honey bee brain genes. The results of this study are helpful to improve the application of RNA interference techniques in honey bees and other insects.

**Abstract:**

RNA interference (RNAi) has been used successfully to reduce target gene expression and induce specific phenotypes in several species. It has proved useful as a tool to investigate gene function and has the potential to manage pest populations and reduce disease pathogens. However, it is not known whether different administration methods are equally effective at interfering with genes in bees. Therefore, we compared the effects of feeding and injection of small interfering RNA (siRNA) on the messenger RNA (mRNA) levels of alpha-aminoadipic semialdehyde dehydrogenase (*ALDH7A1*), 4-coumarate-CoA ligase (*4CL*), and heat shock protein 70 (*HSP70*). Both feeding and injection of siRNA successfully knocked down the gene but feeding required more siRNA than the injection. Our results suggest that both feeding and injection of siRNA effectively interfere with brain genes in bees. The appropriateness of each method would depend on the situation.

## 1. Introduction

RNA interference (RNAi) was first discovered in transgenic plants [1], followed by the discovery of its use to analyze gene function, and its important applications in several insect species [2,3,4]. In honey bees, RNAi has contributed to the understanding of gene functions in caste differentiation [5,6], sex determination [7,8], lifespan [9,10], social behavior [11], learning, and memory [12,13].

The efficiency and stability of RNAi are of great importance when studying gene functions. RNAi application and efficacy remain variable between insect species, life stages, and genes [14]. Small interfering RNA (siRNA) can induce degradation of the complementary messenger RNA (mRNA) of the target gene, reducing the expression levels of the target gene [15]. Quantifying mRNA levels by quantitative real-time polymerase chain reaction (qRT-PCR) has been widely used to characterize the efficiency of RNAi [12,13,14]. Gene knockdown efficacy depends on the transcript level of the target gene, protein turnover rates, and the efficiency of siRNA uptake by organs or cells [15]. Different interference methods have different locations and times of action on target genes. Two common methods used to administer RNAi in honey bees for gene function studies are via feeding or injection [15]. The responses of cells to these two administration methods are considerably different and lead to significant differences in the effectiveness of RNAi treatments [15,16]. For example, the injection of double-stranded RNA (dsRNA) into the body cavity of a locust caused a higher sensitivity than that induced by the feeding of dsRNA [17]. Although the feeding of dsRNA often requires more dsRNA than injection, this method is both less invasive and has a longer-lasting silencing effect in honey bees [18,19]. Both methods have their merits, but, as far as we know, feeding of RNAi has never been used to suppress gene expression in the brains of honey bees. It is thought that gene expression in the bees’ brains can only be knocked down by local injections [20]. However, chemically modified siRNA has been developed that could successfully interfere with brain genes by intravenous injection [21]. We selected *ALDH7A1*, *4CL* and *HSP70* as the target genes, which have been reported to be expressed in honey bees [22]. We also compared the efficiency of feeding and injection of chemically modified siRNA and unmodified siRNA on gene expression in the brains of bees.

*ALDH7A1* is a member of the *ALDH* family and is mainly involved in aldehyde oxidation and aldehyde detoxification [23,24,25]. It affects a large number of neurotransmitters and neurohormones involved in learning, memory, behavior, and energy metabolism [26]. Additionally, *ALDH7A1* may be involved in the regulation of honey bee caste differentiation [22]. The *4CL* gene is involved in p-coumaric acid synthesis in honey bee larval diets and may be involved in honey bee caste differentiation [27,28,29,30]. *HSP70* is a member of the *HSP* family, which protects cells from both biotic and abiotic stress stimuli [31]. It is involved in the regulation of natural bee metabolism, flight behavior, learning, and memory [32,33,34,35]. Therefore, the ability to interfere with the expression of these genes in the honey bee brain is important for future experiments.

Here, we used three genes to study the effect of different siRNA delivery methods on honey bee mortality and gene expression. These data can contribute to better understanding of the importance of siRNA in honey bee RNAi, and inform us about the RNAi methods to be used in different experimental conditions.

## 2. Materials and Methods

### 2.1. Insects

We obtained the honey bees (*Apis mellifera*) for this study from Jiangxi Agricultural University (28.46 μN, 115.49 μE), Nanchang, China, in 2021. A frame of capped brood was removed from a colony and placed in a cage within a 34 °C humidified incubator overnight. Honey bees were collected within eight hours of emergence to ensure that they were the same age. Newly emerged honey bees were kept in a humidified incubator for six days before the experimental treatment began. The honey bees were starved for 3 h prior to injection and feeding of siRNA.

### 2.2. siRNA Preparation and Injection

*ALDH7A1*-specific siRNA (forward: GCAUGGAUUCAAUGGG-CAUTT, reverse: AUGCCCAUUGAAUCCAUGCTT), *HSP70*-specific siRNA (forward: GCUCGAUGCAACCAAUUATT, reverse: UAAUUGGUUAGCAUCGAGCTT) and *4CL*-specific siRNA (forward: GGUGAAAGAUAUGCUAAUATT, reverse: UAUUAGCAUAUCUUUCACCTT) sequences were designed by siDirect (http://sidirect2.rnai.jp/; accessed on 13 September 2022) and DSIR (http://biodev.extra.cea.fr/DSIR/DSIR.html; accessed on 13 September 2022). GenePharma (Shanghai, China; Shanghai Jima Pharmaceutical Technology) helped us synthesize 2′-O-methyl (2′-Ome) modified and unmodified siRNAs. Negative control siRNA is widely used as a control and has no effect on gene expression in bees [36]. Therefore, in this experiment, siRNA-NC (forward: UUCUCCGAACGUGUCACGUTT, reverse: ACGUGACACGUUCGGAGAATT) was used in the control group.

During siRNA injecting, honey bees were tied inside a copper tube and then placed under a stereomicroscope with 1.5 mm sponge double-sided tape under the bee’s brain (to ensure that the bee’s brain did not move around; the sides of the proboscis could also be fastened with a pin). The fluff was scraped from the bee’s brain with a 5 mL syringe needle. The tip of a 5 mL syringe was used to make a crack of about 1 mm in front of the median ocellus. (The tip should not be too deep in the bee’s brain to ensure survival). The siRNA was injected into the bee’s brain through the fissure in the bee’s brain using a microinjector (FemtoJet 4i, Eppendorf; Figure S1a). Honey bees in the experimental group were injected with 1 μL of siRNA solution (siRNA-ALDH7A1, siRNA-4CL, siRNA-HSP70) by microinjector. siRNA solutions were diluted in ddH_2_O to six different concentrations (0.5 μg/μL, 1 μg/μL, 2 μg/μL, 5 μg/μL, 10 μg/μL, and 15 μg/μL). The honey bees in the control group were treated the same as the honey bees in the experimental group, except that the honey bees in the control group were injected siRNA-NC. After the injection, Vaseline was applied to the fissure in the bees’ brains to avoid infection. One hundred honey bees were injected for each group. Fifty of the honey bees were used for survival analysis and the remaining honey bees were used for qRT-PCR.

During siRNA feeding, honey bees in the experimental group were fed 5 μL of siRNA solution (siRNA-ALDH7A1, siRNA-4CL, siRNA-HSP70) by a pipettor (Figure S1b). siRNA solutions were diluted in ddH_2_O to six different concentrations (0.1 μg/μL, 0.2 μg/μL, 0.4 μg/μL, 1 μg/μL, 2 μg/μL, and 3 μg/μL). The honey bees in the control group were treated the same as the honey bees in the experimental group, except that the honey bees in the control group were fed siRNA-NC. If a honey bee could not completely eat all 5 μL of siRNA solution, this honey bee was abandoned. One hundred honey bees were fed for each group. Fifty of the honey bees were used for survival analysis and the remaining honey bees were used for qRT-PCR.

At 8 h, 16 h, 24 h, 48 h, and 72 h after injection and feeding, the brains of the honey bees were dissected, and knockdowns were verified using qRT-PCR.

### 2.3. RNA Preparation and qRT-PCR Assay

Total RNA was extracted from the pooled brains with Trizol (Transgen; Beijing, China), and reverse transcribed to obtain cDNA using the PrimeScript™RT reagent kit (Takara; Tokyo Japan). The obtained cDNA was used for qRT-PCR analysis. The qRT-PCR analysis was performed using the ABI 7500 real-time quantitative PCR system (ABI; Waltham, MA, USA) to detect the expression levels of genes, with *GAPDH* as an internal control. Two bee brains were pooled as a sample. Four biological replicates were performed for each sample, and each biological replicate included three technical replicates. Primers were designed by Premier 5.0 software based on the sequences. The primers for the qRT-PCR assay are provided in Table 1. The cycle threshold value for each sample was obtained by calculating the mean of technical replicates. The data were analyzed by 2^−^^ΔΔCT^. When the *p* value is less than 0.05 on an ANOVA test, it is considered as a significant difference.

### 2.4. Effects of Different Modes of siRNA Delivery on the Survival of Honey Bees

To confirm whether the different ways of delivering siRNA influence the survival of honey bees, honey bees were collected and fed using the previous experimental method. At six days old, the experimental group of honey bees was administered RNAi by the method mentioned in Section 2.2. They were then re-placed in an incubator for rearing (n = 50 per cage). The control group received no treatment. The number of dead honey bees was recorded at 12 noon each day and the dead bees were removed. When all the honey bees were dead, the data were counted and analyzed.

### 2.5. Data Analysis and Statistics

An ANOVA test was conducted to analyze the difference between mRNA levels. The results were expressed as mean ± SE. The Kaplan–Meier method was used to analyze the differences between the control and treatment groups. A value of *p* < 0.05 was considered statistically significant. All statistical data were analyzed with SPSS 25.0 (IBM, New York, NY, USA).

## 3. Results

### 3.1. The Effects of RNA Interference Methods on the Survival of Honey Bees

Recent studies have shown that both injection-induced damage and high doses of the reagent can cause a rapid increase in honey bee mortality. The high mortality rate of bees may have an impact on subsequent experiments. Therefore, we compared the effects of feeding and injecting high concentrations of siRNA on honey bee mortality.

**Figure 1:**
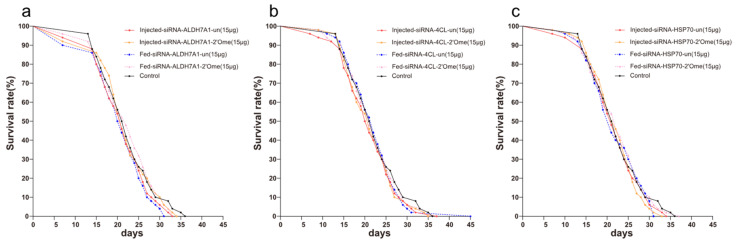
Survival curves of honey bees after the injection and feeding of siRNA. (**a**) Effects of different delivery methods and different modified siRNAs on the survival rate of honey bees. Red solid line represents the injection of unmodified siRNA-ALDH7A1; Orange solid line represents the injection of 2′Ome modified siRNA-ALDH7A1; Blue dashed line represents the feeding of unmodified siRNA-ALDH7A1; Pink dashed line represents the feeding of 2′Ome modified siRNA-ALDH7A1; Black solid line represents honey bees without any treatment. (**b**) siRNA-4CL and (**c**) siRNA-HSP70 were used, otherwise consistent with (**a**). Each group has 50 honey bees.

The different delivery methods, and whether the siRNA was modified or not, had no effect on the survival rate of honey bees (log-rank, chi-square = 2.99, df = 4, *p* = 0.56, Figure 1a; log-rank, chi-square = 0.77, df = 4, *p* = 0.94, Figure 1b; log-rank, chi-square = 0.85, df = 4, *p* = 0.93, Figure 1c). The pairwise comparison between samples is shown in supplementary Table S1.

### 3.2. The Effects of ALDH7A1 RNAi Knockdown on mRNA Levels

**Figure 2:**
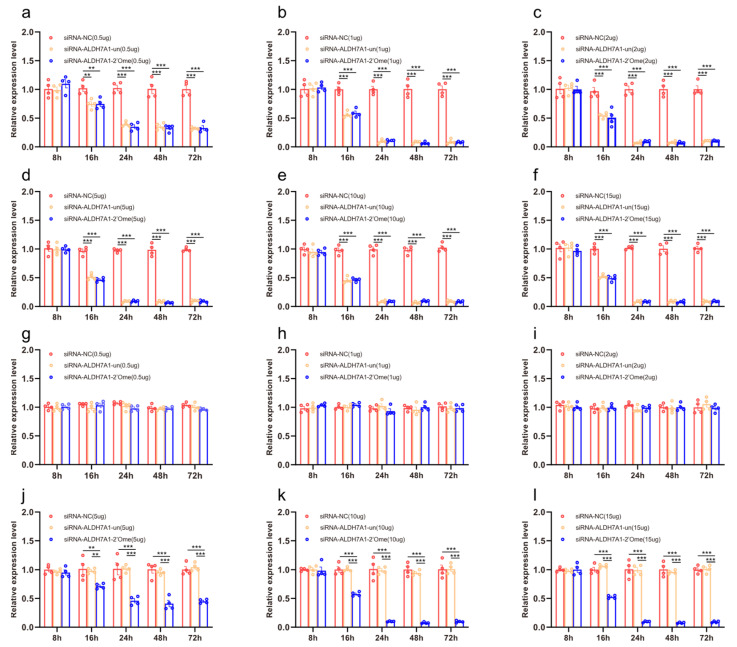
The effects of siRNA on *ALDH7A1* mRNA transcripts in honey bee brains. The mRNA levels of *ALDH7A1* were analyzed at 8 h, 16 h, 24 h, 48 h, and 72 h after injection of 0.5 μg (**a**), 1 μg (**b**), 2 μg (**c**), 5 μg (**d**), 10 μg (**e**), and 15 μg (**f**) siRNA, or after feeding of 0.5 μg (**g**), 1 μg (**h**), 2 μg (**i**), 5 μg (**j**), 10 μg (**k**), and 15 μg (**l**) siRNA. The red column shows the relative expression of siRNA-NC injected. The orange column shows the relative expression of siRNA-ALDH7A1-un injected. The blue column shows the relative expression of siRNA-ALDH7A1-2′Ome injected. Each circle represents a biological repeat. ** indicates *p* < 0.01; *** indicates *p* < 0.001, tested by ANOVA test. Detailed data are shown in Table S2.

We quantified the mRNA transcripts of *ALDH7A1* in the honey bee brains 8 h, 16 h, 24 h, 48 h, and 72 h after administering RNAi using qRT-PCR. At 16 h after the injection, the qRT-PCR results showed that different doses of siRNA reduced the expression of *ALDH7A1*. The effect lasted up to 72 h (Figure 2a–f; Table S2). The injection of 2′Ome modified siRNA (siRNA-ALDH7A1-2′Ome) and unmodified siRNA (siRNA-ALDH7A1-un) had the same effect on the expression of the *ALDH71* gene. Feeding low doses of siRNA-ALDH7A1-un or siRNA-ALDH7A1-2′Ome had no effect on *ALDH7A1* expression (Figure 1g–i). The expression of *ALDH7A1* was affected by feeding high doses of siRNA-ALDH7A1-2′Ome but was not affected by feeding high doses of siRNA-ALDH7A1-un (Figure 2j–l). Both the feeding and injection of 2′Ome modified siRNA successfully reduced *ALDHA71* mRNA (Figure 2; Table S2). However, the knockdown required different siRNA dosages. When injecting siRNA-ALDH7A1-2′Ome, only 1 μg RNA was required to produce the best knockout effect, while 10 μg siRNA-ALDH7A1-2′Ome was required when feeding.

### 3.3. The Effects of 4CL RNAi Knockdown on mRNA Levels

**Figure 3:**
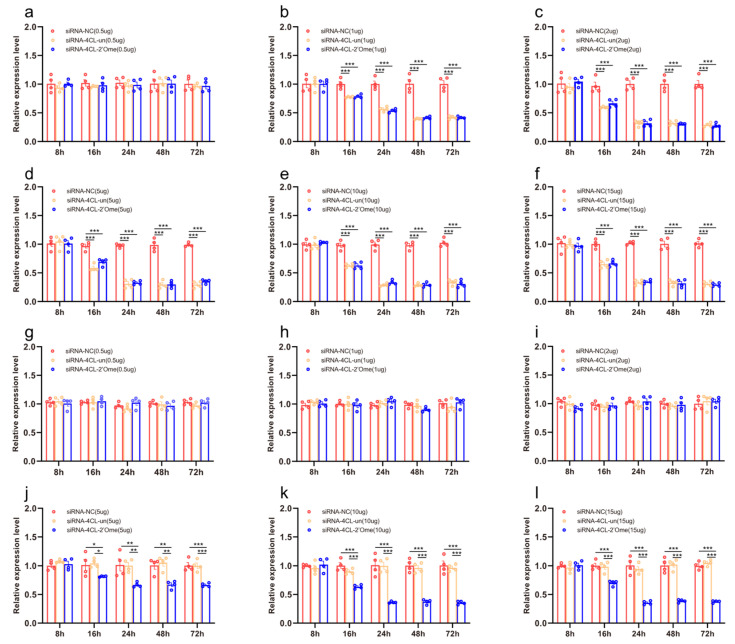
The effects of siRNA on 4CL mRNA transcripts in honey bee brains. The mRNA levels of *4CL* were analyzed at 8 h, 16 h, 24 h, 48 h, and 72 h after injection of 0.5 μg (**a**), 1 μg (**b**), 2 μg (**c**), 5 μg (**d**), 10 μg (**e**), and 15 μg (**f**) siRNA, or after feeding of 0.5 μg (**g**), 1 μg (**h**), 2 μg (**i**), 5 μg (**j**), 10 μg (**k**), and 15 μg (**l**) siRNA. The red column shows the relative expression of siRNA-NC injected. The orange column shows the relative expression of siRNA-4CL-un injected. The blue column shows the relative expression of siRNA-4CL-2′Ome injected. Each circle represents a biological repeat. * Indicates *p* < 0.05, ** indicates *p* < 0.01, *** indicates *p* < 0.001, tested by ANOVA test. Detailed data are shown in Table S3.

We quantified the mRNA transcripts of *4CL* in the honey bee brains 8 h, 16 h, 24 h, 48 h, and 72 h after administering RNAi using qRT-PCR. At 16 h after the injection, the qRT-PCR results showed that all doses of siRNA except 0.5 μg reduced the expression of *4CL*. The effect lasted up to 72 h (Figure 3b–f; Table S3). The injection of 2′Ome modified siRNA (siRNA-4CL-2′Ome) and unmodified siRNA (siRNA-4CL-un) had the same effect on the expression of the *4CL* gene. Feeding low doses of siRNA-4CL or siRNA-4CL-2′Ome had no effect on the gene expression of *4CL* (Figure 1g–i). The expression of *4CL* was affected by feeding high doses of siRNA-4CL-2′Ome but was not affected by feeding high doses of siRNA-4CL-un (Figure 3j–l). Both the feeding and injection of siRNA successfully reduced *4CL* mRNA (Figure 3; Table S3). However, the knockdown required different siRNA dosages. When injecting siRNA-4CL-2′Ome, only 2 μg RNA was required to produce the best knockout effect, while 10 μg siRNA-4CL-2′Ome was required when feeding.

### 3.4. The Effects of HSP70 RNAi Knockdown on mRNA Levels

**Figure 4:**
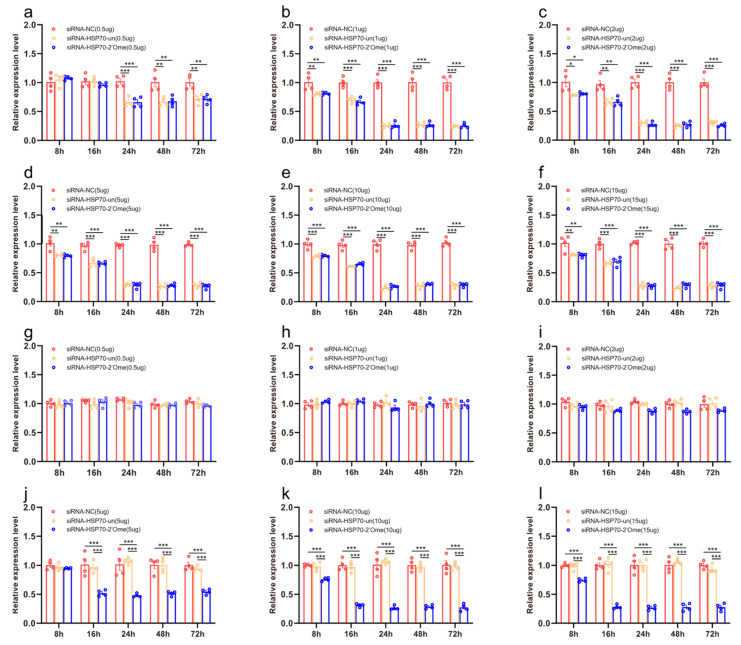
The effects of siRNA on *HSP70* mRNA transcripts in honey bee brains. The mRNA levels of *HSP70* were analyzed at 8 h, 16 h, 24 h, 48 h, and 72 h after injection of 0.5 μg (**a**), 1 μg (**b**), 2 μg (**c**), 5 μg (**d**), 10 μg (**e**), and 15 μg (**f**) siRNA, or after feeding of 0.5 μg (**g**), 1 μg (**h**), 2 μg (**i**), 5 μg (**j**), 10 μg (**k**), and 15 μg (**l**) siRNA. The red column shows the relative expression of siRNA-NC injected. The orange column shows the relative expression of siRNA-HSP70-un injected. The blue column shows the relative expression of siRNA-HSP70-2′Ome injected. Each circle represents a biological repeat. * Indicates *p* < 0.05, ** indicates *p* < 0.01, *** indicates *p* < 0.001, tested by ANOVA test. Detailed data are shown in Table S4.

We quantified the mRNA transcripts of *HSP70* in the honey bee brains 8 h, 16 h, 24 h, 48 h, and 72 h after administering RNAi using qRT-PCR. At 8 h after the injection, the qRT-PCR results showed that different doses of siRNA reduced the expression of *HSP70*. The effect lasted up to 72 h (Figure 4a–f; Table S4). The injection of 2′Ome modified siRNA (siRNA-HSP70-2′Ome) and unmodified siRNA (siRNA-HSP70-un) had the same effect on the expression of the *HSP70* gene. Feeding low doses of siRNA had no effect on the gene expression of *HSP70* (Figure 4g–i). The expression of *HSP70* was affected by feeding high doses of siRNA-HSP70-2′Ome but was not affected by feeding high doses of siRNA-HSP70-un (Figure 4j–l). Both the feeding and injection of siRNA successfully reduced *HSP70* mRNA (Figure 4; Table S4). However, the knockdown required different siRNA dosages. When injecting siRNA-HSP70-2′Ome, only 1 μg RNA was required to produce the best knockout effect, while 10 μg siRNA-HSP70-2′Ome was required when feeding.

## 4. Discussion

In this study, we investigated the efficiency of gene knockdown using different dosages and different administration methods (via injection and feeding) in honey bee brains at mRNA levels. We determined the optimal time-window and dosage for studying the functions of the honey bee brain using RNAi.

In the experiment using the siRNA injection, we compared the efficacy of different RNAi molecules. Although the initial time of effect is different, they can effectively reduce the expression of target genes, and the effect can last for at least 48 h. In general, the knockdown effect is also different in different target genes of the same organism, depending on the specific structure of the siRNA and the molecular dose of RNAi [37]. Several researches have indicated that there is also great variability in the knockdown effect of siRNA due to the different regions of the targeted gene [20,38]. This would explain why *HSP70* expression began to decline as early as 8 h after siRNA injection. However, the expression of *ALDH7A1* and *4CL* did not begin to decrease until 16 h later. Moreover, the expression of *HSP70* was decreased by injection of 0.5 μg siRNA, while *ALDH7A1* and *4CL* needed an injection of 1 μg siRNA.

Although modified siRNA has been shown to interfere with brain genes by injection in mammals [39,40], the feeding of siRNA has not been previously reported to inhibit gene expression in honey bee brains [41]. This may be due to the low accumulation and poor stability of siRNA in the brain. Researchers are working on ways to deliver siRNA systematically, efficiently, and safely to the brain. Presently, there are two main methods. Firstly, siRNA can be encapsulated in nanoparticles to avoid degradation. It interacts with cell-surface receptors expressed in the brain to provide cell uptake of siRNA. Alternatively, siRNA can be chemically modified so that it can enter specific tissues [21,42,43,44]. Our results clearly show that feeding 2′Ome-modified siRNA can reduce gene expression in the honey bee brain, whereas unmodified siRNA cannot.

We found no difference in honey bee mortality between the siRNA-treated bees and the control group. This indicated that neither of the siRNA administration methods affected the survival rate of the honey bees. When siRNA was injected, only 1 μg was required to achieve the highest level of interference, while 10 μg was required when feeding siRNA. This suggests that the effect of gene knockdown depends on the delivery mode and dose of siRNA, which is consistent with Mittal’s view [37].

Injection and feeding each have their advantages and disadvantages. Injection has an important advantage in that it allows researchers to deliver the siRNA immediately to the tissue or into the hemolymph and hence avoiding possible barriers such as the blood-brain barrier or the gut epithelium which could be a problem in feeding. Another advantage is that the exact amount of dsRNA brought into an organism is known, in contrast to delivery by soaking or in some cases by feeding. However, this method has some disadvantages. The work itself is more delicate than other methods. Factors such as the choice of needle, the angle of the injection, and the volume and position of the injection are all very important and vary greatly between organisms. For example, in *Acyrthosiphon pisum*, the volume of the injection has been reported to be critical to the survival of the aphid after injection [45]. Damage to the cuticle caused by the injection may stimulate immune function, which could further complicate the interpretation of the results [46,47].

Feeding also has many advantages. It is easy to manipulate, convenient, and causes less damage to the insect [48,49]. It also has advantages in small insects, which are harder to manipulate using microinjections [49,50]. This method is also very suitable for the screening of pest control genes [47]. However, feeding is not suitable for all species. For example, the dsRNA designed for *Spodoptera litura* did not succeed in disrupting the target [51]. Sometimes feeding is less effective than injection, such as in *Caenorhabditis elegans* [52] and *Rhodnius prolixus* [50]. In addition, the RNAi efficiency of siRNA ingestion in different species may vary depending on the intestinal environment. Another limitation of siRNA feeding is the difficulty of determining the amount of siRNA that enters the insect through ingestion, which may affect many investigations. In addition, from the results of this article, to interfere with gene expression in the honey bees’ brain by feeding, modified siRNA must be used. Therefore, the appropriateness of the injection compared to the feeding of siRNA needs to be decided based on the requirements of each experiment.

## 5. Conclusions

The results showed that the injection of unmodified or 2′Ome-modified siRNA could reduce the expression of honey bee brain genes. However only feeding 2′Ome-modified siRNA could reduce the expression of bee brain genes. Feeding unmodified siRNA did not reduce gene expression in the bee brain. siRNA feeding and siRNA injection had no significant effect on honey bee mortality, but less siRNA was required for siRNA injection.

## Figures and Tables

**Table 1:** Primer sequences for quantitative qRT-PCR.

Genes	Forward Primer	Reverse Primer	Length
*ALDH7A1*	GATGGGTCCTCTTGGTTCAG	TATAGTGGCACGTCGCATGT	157
*HSP70*	GATTCGCAAAGGCAAGCTAC	CCGCTGTTGACTTCACTTCA	217
*4CL*	CAAGTGGACCTTTCGTGGTT	TCTTGTGCGTCAACATGACA	198
*GAPDH*	GCTGGTTTCATCGATGGTTT	ACGATTTCGACCACCGTAAC	180

## Data Availability

The data presented in this study are available in this article.

## Supplementary Materials

The following supporting information can be downloaded at: https://www.mdpi.com/article/10.3390/insects13100928/s1, Figure S1. (a) Schematic diagram of honey bee injection. The green arrow shows the microinjector used to inject the bees with siRNA. (b) Schematic diagram of honey bee feeding. The green arrow shows the pipettor used to inject the bees with siRNA. Table S1: Pairwise comparison of the effects of different delivery methods and different modified siRNA methods on honey bee mortality. Table S2: Effects of different delivery methods and different modified siRNA methods on the expression of *ALDH7A1* in honey bees. Table S3: Effects of different delivery methods and different modified siRNA methods on the expression of *4CL* in honey bees. Table S4: Effects of different delivery methods and different modified siRNA methods on the expression of *HSP70* in honey bees.
