# Supplementary material for: A Comparison of RNA Interference via Injection and Feeding in Honey Bees
Source: Insects. 2022 Oct 13;13(10):928. doi: 10.3390/insects13100928 (PMC9604379; doi:10.3390/insects13100928)
Supplement: Supplementary file 1 [file insects-13-00928-s001.zip › supplementary.pdf]

a b

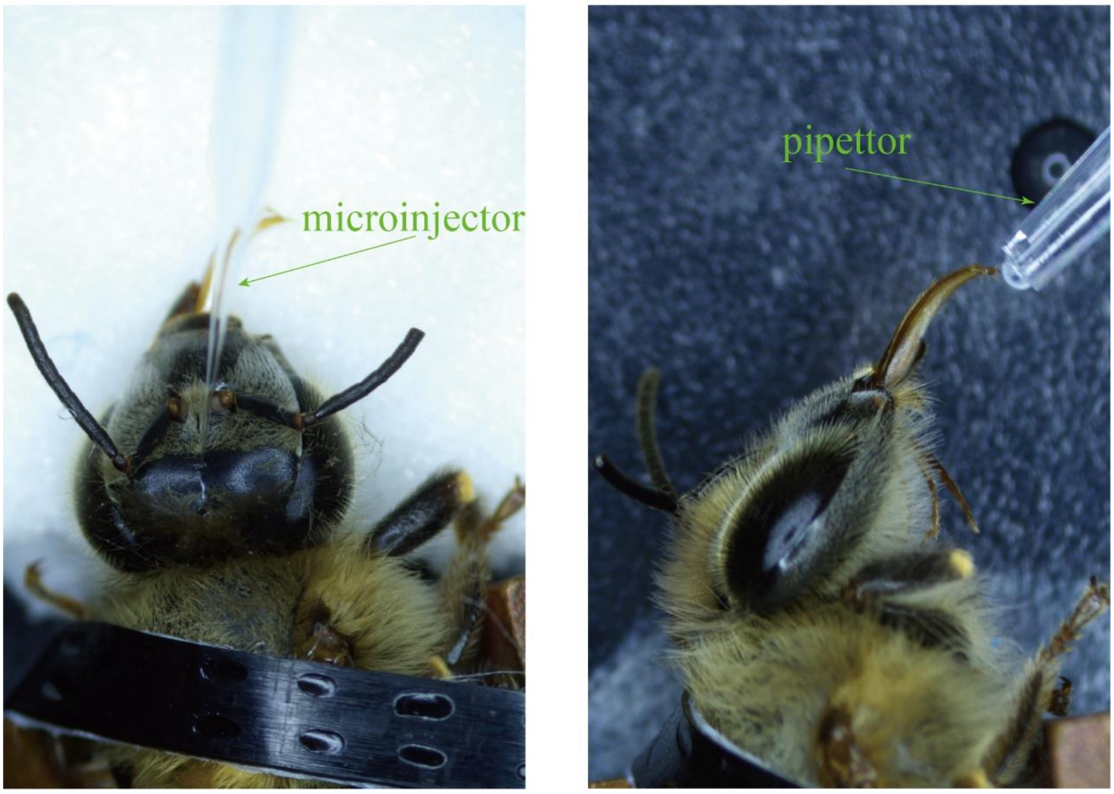

Figure S1. (A) Schematic diagram of honey bee injection. The green arrow shows the microinjector used to inject the bees with siRNA. (B) Schematic diagram of honey bee feeding. The green arrow shows the pipettor used to inject the bees with siRNA.
